# The impact of chemo- and radiotherapy treatments on selfish *de novo FGFR2* mutations in sperm of cancer survivors

**DOI:** 10.1093/humrep/dez090

**Published:** 2019-07-26

**Authors:** Geoffrey J Maher, Marie Bernkopf, Nils Koelling, Andrew O M Wilkie, Marvin L Meistrich, Anne Goriely

**Affiliations:** 1 Clinical Genetics Group, MRC-Weatherall Institute of Molecular Medicine, University of Oxford, Oxford, UK; 2 Nuffield Division of Clinical Laboratory Sciences, Radcliffe Department of Medicine, University of Oxford, Oxford, UK; 3 Department of Experimental Radiation Oncology, University of Texas M.D. Anderson Cancer Center, Houston, USA

**Keywords:** mutation rate, cancer treatment, chemotherapy, radiation therapy, spermatogonial stem cells, recovery of fertility, *de novo* mutations, Apert syndrome

## Abstract

**STUDY QUESTION:**

What effect does cancer treatment have on levels of spontaneous selfish fibroblast growth factor receptor 2 (*FGFR2)* point mutations in human sperm?

**SUMMARY ANSWER:**

Chemotherapy and radiotherapy do not increase levels of spontaneous *FGFR2* mutations in sperm but, unexpectedly, highly-sterilizing treatments dramatically reduce the levels of the disease-associated c.755C > G (Apert syndrome) mutation in sperm.

**WHAT IS KNOWN ALREADY:**

Cancer treatments lead to short-term increases in gross DNA damage (chromosomal abnormalities and DNA fragmentation) but the long-term effects, particularly at the single nucleotide resolution level, are poorly understood. We have exploited an ultra-sensitive assay to directly quantify point mutation levels at the *FGFR2* locus.

**STUDY DESIGN, SIZE, DURATION:**

‘Selfish’ mutations are disease-associated mutations that occur spontaneously in the sperm of most men and their levels typically increase with age. Levels of mutations at c.752–755 of *FGFR2* (including c.755C > G and c.755C > T associated with Apert and Crouzon syndromes, respectively) in semen post-cancer treatment from 18 men were compared to levels in pre-treatment samples from the same individuals (n = 4) or levels in previously screened population controls (n = 99).

**PARTICIPANTS/MATERIALS, SETTING, METHODS:**

Cancer patients were stratified into four different groups based on the treatments they received and the length of time for spermatogenesis recovery. DNA extracted from semen samples was analysed using a previously established highly sensitive assay to identify mutations at positions c.752–755 of *FGFR2*. Five to ten micrograms of semen genomic DNA was spiked with internal controls for quantification purposes, digested with MboI restriction enzyme and gel extracted. Following PCR amplification, further MboI digestion and a nested PCR with barcoding primers, samples were sequenced on Illumina MiSeq. Mutation levels were determined relative to the spiked internal control; in individuals heterozygous for a nearby common single nucleotide polymorphism (SNP), mutations were phased to their respective alleles.

**MAIN RESULTS AND THE ROLE OF CHANCE:**

Patients treated with moderately-sterilizing alkylating regimens and who recovered spermatogenesis within <3 years after therapy (Group 3, n = 4) or non − alkylating chemotherapy and/or low gonadal radiation doses (Group 1, n = 4) had mutation levels similar to untreated controls. However, patients who had highly-sterilizing alkylating treatments (i.e. >5 years to spermatogenesis recovery) (Group 2, n = 7) or pelvic radiotherapy (Group 4, n = 3) exhibited c.755C > G mutation levels at or below background. Two patients (A and B) treated with highly-sterilizing alkylating agents demonstrated a clear reduction from pre-treatment levels; however pre-treatment samples were not available for the other patients with low mutation levels. Therefore, although based on their age we would expect detectable levels of mutations, we cannot exclude the possibility that these patients also had low mutation levels pre-treatment. In three patients with low c.755C > G levels at the first timepoint post-treatment, we observed increasing mutation levels over time. For two such patients we could phase the mutation to a nearby polymorphism (SNP) and determine that the mutation counts likely originated from a single or a small number of mutational events.

**LIMITATIONS, REASONS FOR CAUTION:**

This study was limited to 18 patients with different treatment regimens; for nine of the 18 patients, samples from only one timepoint were available. Only 12 different *de novo* substitutions at the *FGFR2* c.752–755 locus were assessed, two of which are known to be disease associated.

**WIDER IMPLICATIONS OF THE FINDINGS:**

Our data add to the body of evidence from epidemiological studies and experimental data in humans suggesting that male germline stem cells are resilient to the accumulation of spontaneous mutations. Collectively, these data should provide physicians and health-care professionals with reassuring experimental-based evidence for counselling of male cancer patients contemplating their reproductive options several years after treatment.

**STUDY FUNDING/COMPETING INTEREST(S):**

This work was primarily supported by grants from the Wellcome (grant 091182 to AG and AOMW; grant 102 731 to AOMW), the University of Oxford Medical Sciences Division Internal Fund (grant 0005128 to GJM and AG), the National Institute for Health Research (NIHR) Oxford Biomedical Research Centre Programme (to AG) and the US National Institutes of Health (to MLM). The funders had no role in study design, data collection and analysis, decision to publish, or preparation of the manuscript. None of the authors has any conflicts of interest to declare.

**TRIAL REGISTRATION NUMBER:**

NA

## Introduction

Over the past few decades, improved cancer treatments have led to a dramatic increase in patient survival rates. However, infertility is a common and undesirable side-effect of chemotherapy and radiotherapy. In men, it is well-established that the risk of infertility is treatment and dose-dependent: while treatment with lower dose radiation or chemotherapy may lead to transient oligozoospermia or azoospermia (Howell and Shalet 2005), alkylating agents and high gonadal levels of radiation (>2 Gy) have the strongest gonadotoxic impact on the germ cells and/or supporting somatic cells of the testis, typically resulting in prolonged or even permanent azoospermia (Howell and Shalet 2005, Meistrich 2013).

For men whose spermatogenesis recovers after treatment, the long-term effect on the genetic integrity of their gametes and the associated risks to their future offspring are a common concern. Although semen cryopreservation is recommended prior to treatment (Pacey and Eiser 2011), not all male cancer patients have access to, or choose to use, such facilities. Moreover even patients who have stored semen are unlikely to return to their stored samples once their fertility recovers (Saito *et al.* 2005).

Several studies have attempted to establish the long-term impact of cancer treatments on reproductive safety. However, depending on the approach followed, some have led to conflicting conclusions, making it difficult for physicians to provide evidence-based counselling to cancer survivors. While the majority of epidemiological studies suggest no significant increased risk for offspring of men who have survived cancer (Meistrich and Byrne 2002, Tang *et al.* 2016), experimental data from animal models showed that chemotherapy and radiotherapy can lead to DNA damage in germ cells and have described an increased incidence of congenital malformations in offspring (Kirk and Lyon 1984, Witt and Bishop 1996, Generoso *et al.* 1984, Adewoye *et al.* 2015). Analysis of human sperm provides another direct experimental approach, but due to the technical difficulty in identifying rare spontaneous DNA lesions, studies so far have mainly focused on gross DNA damage, for example chromosomal abnormalities and DNA fragmentation: both parameters increase in the first few months post-treatment, but return to baseline within 18–24 months (Frias *et al.* 2003, Martinez *et al.* 2017). As a result, patients are generally advised to wait >2 years after treatment before attempting to father a child (Choy and Brannigan 2013).

Recently, using whole-genome sequencing (WGS) of family trios, Kryukov et al (Kryukov *et al.* 2016) have shown that the number of *de novo* point mutations in children conceived by two men post-testicular cancer treatment (three cycles of bleomycin, etoposide, and cisplatin) did not differ significantly from their siblings fathered pre-diagnosis (Kryukov *et al.* 2016). Although there was a marginal increase in *de novo* mutations in the children conceived after therapy, this could be accounted for by the well-documented linear increase due to advancing paternal age (Kong *et al.* 2012). Similarly, a WGS study of a non-Hodgkin lymphoma patient treated with radiotherapy and 12 soldiers working on equipment that was emitting high-frequency radiation found that the *de novo* point mutation load in their offspring was comparable to that of controls, although there appeared to be an increase in tandem and clustered point mutations (Holtgrewe *et al.* 2018). Overall these reports, which suggest that cancer treatment has little long-term overall mutagenic effect on the male germline, should be reassuring, but it is important to recognise that the available studies remain small in scale, have a low resolution to quantify point mutations, and have only assessed the impact of a few treatment regimens.

**Figure 1:**
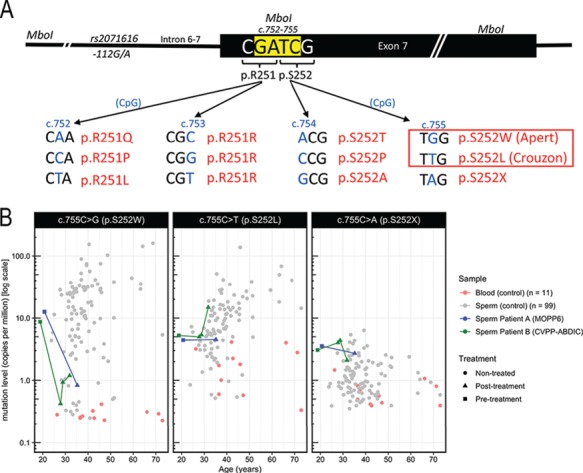
**Genomic context of the *FGFR2* c.752–755 locus and mutation levels measured at *FGFR2* c.755 in controls and patients.** **(A)** Illustration of the 12 substitutions in *fibroblast growth factor receptor 2* (*FGFR2)* (and encoded protein changes) enriched by *Mbo*I digestion (*Mbo*I site in yellow) that were analysed in this study. The known pathogenic mutations are boxed. **(B)** Mutation levels in sperm of patients A (blue) and B (green) are compared to those of a cohort of 11 blood (red) and 99 sperm (grey) samples from controls previously quantified using the same Pyrosequencing methodology (Goriely *et al.* 2003).

Although typical/neutral *de novo* mutations cannot be assessed in DNA extracted from semen samples owing to the rarity of such spontaneous events and their anticipated ultra-low frequency (~1.2 x 10^−8^ per nucleotide per generation) (Kong *et al.* 2012), so-called ‘selfish’ mutations may be present 2–4 orders of magnitude more abundantly and can be directly quantified in sperm using sensitive locus-specific assays (Goriely *et al.* 2003, Goriely *et al.* 2009, Giannoulatou *et al.* 2013). Such mutations confer a selective advantage to the spermatogonial stem cells (SSCs) in which they arise, resulting in their clonal expansion (Goriely *et al.* 2003, Qin *et al.* 2007, Maher *et al.* 2016), through a mechanism akin to oncogenesis. Over time this process of ‘selfish spermatogonial selection’, which occurs in the testes of all men as they age, results in elevated mutation levels in sperm (Goriely *et al.* 2003, Goriely *et al.* 2009, Giannoulatou *et al.* 2013). To date, all reported selfish mutations occur in growth factor receptors [fibroblast growth factor receptor 2 and 3 (*FGFR2*, *FGFR3)*, *RET*] and members of the RAS/mitogen activated protein kinase (RAS/MAPK) signaling pathway and are identical or allelic to oncogenic mutations identified in tumor samples (Goriely and Wilkie 2012, Maher *et al.* 2018). The best-studied locus is *FGFR2* c.755C at which the germline c.755C > G mutation (p.S252W) causes Apert syndrome and the c.755C > T mutation (p.S252L) has been associated with mild Crouzon syndrome (Fig. 1A). These variants have also been described as somatic driver mutations associated with endometrial cancer (Dutt *et al.* 2008) or other tumor types (COSMIC v87; https://cancer.sanger.ac.uk/cosmic).

Hence in order to provide further evidence and directly assess the genetic disease risk for selfish mutations in the future offspring of cancer survivors, we have exploited our ability to quantify 12 different *de novo* mutations at the *FGFR2* c.752–755 locus (Fig. 1A). Here we assay sperm samples from 18 cancer survivors to assess the effects of several different cancer treatment types on levels of both disease-associated and neutral mutations at this locus in human sperm.

## Materials and Methods

### Sample collection and preparation

Samples were collected as part of several longitudinal studies of the effects of different cancer therapy regimens on sperm count, quality, and genetic integrity. The studies were approved by the Institutional Review Board of the University of Texas M. D. Anderson Cancer Center in accordance with an assurance document filed with and approved by the Department of Health and Human Services. Pre-treatment samples were either obtained from patients who declined semen cryopreservation or retrieved from a sperm bank after the patient recovered spermatogenesis. Post-treatment samples were obtained at follow-up clinic visits, collected by masturbation, and frozen within 3 hours after ejaculation. Verbal informed consent, as required by the Laboratory Protocols, was obtained from all participants. Long-term storage of all samples was at −80 °C. Samples from non-treated controls were collected with the permission of the Oxfordshire Research Ethics Committee (OxREC C03.076) and written informed consent was obtained. For individuals with more than one sample, sample relationships were confirmed by genotyping of common single nucleotide polymorphisms (SNPs) (data not shown).

Some of the sperm count data of the patients on this study have been included in earlier publications in which treatment regimens are described in more detail (see also Supplementary Table SI): MOPP (nitrogen mustard, Oncovin, procarbazine (PCZ), prednisone) (two or six cycles with and without pelvic radiotherapy (XRT)) (da Cunha *et al.* 1984); CYADIC (cyclophosphamide (CP), Doxorubicin/Adriamycin (DOX), dacarbazine) (Meistrich *et al.* 1992); CHOP-B (CP, DOX, Oncovin, prednisone, bleomycin) (Pryzant *et al.* 1993); NOVP+radiation (Novantrone, Oncovin, Velban, Prednisone, XRT) (Meistrich *et al.* 1997, Dubey *et al.* 2000); hemipelvic radiation (May *et al.* 2000); CVPP-ABDIC (CP, vincristine, PCZ, prednisone, DOX, bleomycin, dacarbazine, chloroethyl-cyclohexyl-nitrosourea (lomustine)) (Preti *et al.* 1994, Meistrich 2013). For treatments involving alkylating agents, the cyclophosphamide equivalent dose (CED) score was calculated (Supplementary Table SI) according to the formula described in Green *et al.* (2014). We note that while Doxorubicin has been previously shown to increase the risk of azoospermia in patients and have additive effects to cyclophosphamide (Meistrich *et al.* 1990, Meistrich *et al.* 1992, Pryzant *et al*. 1993), it is not considered in the CED formula. As all patients treated with alkylating agents had high CED scores, they were further subdivided into two groups (Group 2 and Group 3) based on their specific treatments (Supplementary Table SI). The time to spermatogenesis recovery was used as an estimate for the gonadotoxic impact of their specific treatment regimens (Supplementary Fig. S1). Detailed sperm counts and collection time points for each patient are given in Supplementary Table SII.

For patients C and G, multiple samples were pooled to have sufficient cell numbers for extraction. Genomic DNA (gDNA) was extracted from all semen samples using the phenol chloroform method previously reported (Goriely *et al.* 2003).

### Mutation assay

DNA concentration from samples of patients A to R was determined using the Qubit fluorometer (Thermo Fisher Scientific, Waltham, MA, USA). A proof of principle study (patients A-B) was performed with 10 μg of gDNA using Pyrosequencing, as described in Goriely *et al.* (2003). For patients C to R and controls, 5 μg of input gDNA was utilized; mutation levels were quantified using Illumina MiSeq 300v2 sequencing (Illumina, San Diego, CA, USA). Mutation levels (copies per million) were quantified relative to spiked-in mutant gDNA carrying the c.755_757delCGCinsTCT mutation in *FGFR2* (‘triple spike’—individual ‘GR’ in (Goriely *et al.* 2003)) at 3 x 10^−5^. gDNA carrying the c.755_756delCGinsTC mutation in *FGFR2* (‘double spike’ in (Goriely *et al.* 2005)) was also spiked in 10^−5^ as an internal control. Briefly, the following was performed for all samples. Spiked DNA samples were digested with 20 U *Mbo*I restriction enzyme (Promega, Madison, WI, USA) for 2 hours at 37 °C, followed by addition of a further 20 U *Mbo*I and 2 hours digestion at 37 °C. Digested samples were run on a 1% Ultrapure Agarose gel (Invitrogen, Carlsbad, CA, USA) at 4 °C overnight and gel extracted using a Zymoclean Gel DNA recovery kit (Zymo Research, Irvine, CA, USA). The locus was amplified with the following PCR primers (5′-3′): Forward TTACAAGGGTCGCGCTCCAGCAGTCTCC and reverse GGGGCTGGGCATCACTGTAAACCTTG using Platinum Pfx DNA Polymerase (Thermo Fisher Scientific) followed by a digestion with 20 U *Mbo*I for 2 hours at 37 °C. For patients A and B, a nested PCR using biotinylated primers (5′-3′) (Forward ATTCATGGGGCCACAGTGTTATTTCAAAGG and reverse AACCTTGCAGACAAACTCTACGTCTCC) was performed in triplicate and products were analyzed by Pyrosequencing (PSQ-96 system, Qiagen, Hilden, Germany) as per (Goriely *et al.* (2003)). For patients C to R and controls, samples were amplified in triplicate using DreamTaq polymerase (Thermo Fisher Scientific) and primers with the same template-specific sequence with additional common sequence tags on the 5′ end (Forward TACGGTAGCAGAGACTTGGTCTATTCATGGGGCCACAGTGTTATTTCAAAGG and reverse ACACTGACGACATGGTTCTACAAACCTTGCAGACAAACTCTACGTCTCC) in a reaction which also contained Access Array Illumina barcoding primers targeting CS (Fluidigm, San Francisco, CA, USA). Barcoded amplicons were pooled, gel extracted with Zymoclean Gel DNA recovery kit (Zymo Research), purified with Axyprep beads (Axygen, Union City, CA, USA) and were sequenced on Illumina MiSeq (300v2 kit). Details of all primers are available in Supplementary Table SIII.

### Mutation quantification

Pyrosequencing analysis was performed as previously described (Goriely et al. 2003). In brief, the Pyrosequencing primer was annealed to the biotinylated PCR product and sequencing with the nucleotide dispensation order G-G-T-G-A-G-C-G-C-T-C-G-T-C-T-C-A-C was performed. Peak heights were recorded using PSQ96 SQA software (Qiagen). Mutation levels were quantified relative to the triple spike mutation (at 3 x 10^−5^). For samples analysed by Illumina MiSeq sequencing, reads were aligned to the human genome (hg19) and using BWA-MEM (Li 2013). Reads with a minimum mapping quality of Q20 were processed with a custom R script (available on request) providing counts for reference and all non-reference sequences at positions c.751–756 of *FGFR2* (chr10:123279675–123279680 (hg19)). Variants were phased to a nearby SNP (rs2071616, chr10:123279795). Sample quality control (QC) was performed as follows: samples with less than 100 triple spike reads were excluded and samples with low or high double spike counts (not within a 2.5-fold range of expected) were excluded. As sufficient *Mbo*I digestion of the wildtype sequence is required to enrich for mutations at the c.752–755 sites, samples with >25% wildtype reads were excluded from further analysis. For the four control samples and 26 patient samples passing QC, mutation levels were quantified relative to the triple spike mutation (at 3 x 10^−5^).

## Results

To study the impact of chemotherapy and radiotherapy on mutation levels in male germ cells, we initially used a previously established highly-sensitive and robust protocol to detect and quantify (using pyrosequencing) mutation levels at positions c.752–755 of *FGFR2* at levels down to 10^−6^ (Goriely *et al.* 2003, Goriely *et al.* 2005). This assay was applied, as a proof-of-principle, to pre- and post-treatment semen samples from two Hodgkin’s lymphoma patients (A and B, Table I), each of whom underwent treatment with six cycles of alkylating agents (MOPP or CVPP-ABDIC, respectively) (Supplementary Table SI). Levels of the mutation (c.755C > G [p.S252W]—Apert syndrome; Fig. 1A) conferring the strongest selective/clonal advantage to SSCs in both patients’ pre-treatment samples were within the normal range (12.6 and 8.7 copies per million [cpm]) when compared to those of a previously published cohort of untreated individuals of different ages (Goriely *et al.* 2003) (Fig. 1B, left panel; Table I). However, in contrast to the population trend of increasing c.755C > G mutation level with age (Goriely *et al.* 2003), in the post-treatment samples (collected ~15 years and ~9 years later for patients A and B, respectively), mutation levels were ~10-fold lower than pre-treatment, at levels close to the assay background (<1 cpm detected in blood). However, Patient B did show a minor increase in levels of c.755C > G mutations at 9.5 and 12.6 years post-treatment. The c.755C > T substitution that encodes p.S252L (mild Crouzon syndrome) confers a weaker selective advantage (Goriely *et al.* 2003, Giannoulatou *et al.* 2013); note that as this C > T transition occurs at a CpG dinucleotide the assay background is higher than that for c.755C > G. While pre-treatment samples and initial post-treatment samples from both patients were less than 10 cpm (similar to background levels in blood), at 12.6 years post-therapy levels of the c.755C > T mutation for patient B had increased to 15 cpm (Fig. 1B, middle panel). The c.755C > A substitution (p.S252*—never reported so far in humans) (Fig. 1B, right panel) and all substitutions at c.752–754, which are not anticipated to be under selection, were at background levels that did not change over time (Supplementary Table SIV).

**Table I:** Patient details for male survivors of cancer and sample details

Patient ID	Sample ID	Treatment	XRT (Gy)	Sperm count (million per ml)	Years to 5 million sperm per ml	Years post treatment	Age (years)	Mean c.755C > G copies per million (± SEM)#	Mean c.755C > T copies per million (± SEM)#	Number of replicates
Group 1 - Non-alkylating and/or hemi- or non-pelvic radiotherapy										
O	O2	NOVP; Abdominal XRT	0.35	30	~1.1	1.5	26.3	9.3 (± 1.7)	3.3 (± 1.4)	5
P	P1	Pre		46		0.0	30.4	9.2 (± 2.5)	1.1 (± 0.6)	3
P	P4	NOVP; Abdominal spade XRT	0.65	38	~0.6	0.8	31.7	14.2	2.0	1
Q	Q1	Pre		123		0.0	39.2	64.9 (± 18.2)	37.1 (± 14.7)	3
Q	Q4	Hemipelvic XRT	0.55	99	0.9	0.1	39.3	29.0 (± 4.4)	32.7 (± 5.9)	2
Q	Q2	“		20		1.0	40.3	156.2 (± 27.9)	21.2 (± 5.4)	4
R	R1	Pre		209		0.0	32.9	4.6 (± 2.9)	12.3 (± 2.1)	4
R	R4	Hemipelvic XRT	0.63	179	0.8	0.1	33.0	5.3 (± 2.2)	7.1 (± 1.9)	2
R	R2	“		73		1.0	33.9	5.9	8.6	1
Group 2 - Highly sterilizing alkylating treatment										
A	A0	Pre		100		0.0	20.7	12.6	4.4	1
A	A1	MOPP6	0	87	~8.3	14.9	35.2	0.8	4.5	1
B	B0	Pre		168		0.0	18.8	8.7	5.2	1
B	B1	CVPP-ABDIC	<0.05	110	<8.5	8.5	27.8	0.4	5.0	1
B	B2	“		127		9.5	28.8	0.9	5.4	1
B	B3	“		380		12.6	31.8	1.2	14.8	1
C	C1/2/3*	CVPP-ABDIC	<0.05	3	~12.9	12.2	39.5	0.1	2.0	1
D	D1	CVPP-ABDIC	<0.05	14	~5.0	5.2	31.1	0.1	1.1	1
D	D2	“		5		6.2	32.1	0.1	4.6	1
E	E0	CVPP-ABDIC	<0.05	27	~6.7	12.1	39.8	0.2 (± 0.1)	4.4 (± 1.4)	3
E	E1	“		65		14.3	41.9	0.1 (± 0.1)	2.4 (± 1.2)	3
E	E2	“		42		15.2	42.8	0.1 (± 0.0)	3.9 (± 1.0)	2
F	F1	CVPP-ABDIC	0	102	(2.7–8.7)	8.6	29.9	0.0	7.8	1
G	G1/2*	CYADIC + Ifosfamide	0	5*	6.9	7.0	46.7	0.1	2.5	1
Group 3 - Moderately sterilizing alkylating agent treatment										
H	H1	CHOP-B/COP-B	<0.05	160	<2.6	2.6	28.4	45.9 (± 4.8)	8.8 (± 4.3)	4
I	I1	MCOP/CMED/HOAP-B	0	14	~1.8	1.9	20.7	0.1 (± 0.0)	7.0 (± 2.2)	2
J	J1	CHOP-B/OAP-B	0	54	~1.8	3.0	33.0	25.8 (± 4.8)	31.3 (± 7.4)	3
K	K1	CHOP-B	<0.05	39	<3.0	3.0	22.6	0.0 (0.0)	2.3 (± 1.1)	4
Group 4 - Pelvic radiotherapy										
L	L1	MOPP3; Pelvic XRT	4.15	87	~7.2	22.0	37.8	0.8	6.1	1
M	M1	MOPP2; Pelvic XRT	3.11	26	<5	6.0	28.5	0.1 (± 0.0)	2.7 (± 0.7)	3
M	M2	“		40		14.0	36.5	11.0 (± 3)	3.4 (± 1.0)	4
N	N1	NOVP; Pelvic XRT	1.85	15	2.1	2.2	33.7	1.4 (± 1.2)	5.1 (± 0.4)	3
N	N2	“		52		4.2	35.7	32.9 (± 8.1)	1.9 (± 0.7)	4

^*^For patients C and G, multiple samples were pooled to have sufficient cell numbers for extraction. Sperm counts and times are the means of values for these pooled samples.

#Detailed mutation levels for each substitution at *fibroblast growth factor receptor 2* (*FGFR2)* c.752–755 are given in Supplementary Table SII. Further information about specific treatments is supplied in Supplementary Table SI.

XRT: Radiotherapy; Gy: Gray. NOVP: Novantrone, Oncovin, Velban, Prednisone, MOPP: nitrogen mustard, Oncovin, procarbazine (PCZ), prednisone (two or six cycles with and without pelvic XRT), CVPP-ABDIC: cyclophosphamide (CP), vincristine, PCZ, prednisone, DOX (Doxorubicin/Adriamycin), bleomycin, dacarbazine, chloroethyl-cyclohexyl-nitrosourea (lomustine), CYADIC: CP, DOX, dacarbazine, CHOP-B: CP, DOX, Oncovin, prednisone, bleomycin, MCOP: Mitoxantrone [Novantrone], CP, Oncovin, prednisolone, CMED: CP, methotrexate, etoposide, dexamethasone), HOAP-B: DOX, Oncovin, Cytarabine, prednisone, bleomycin.

To study further samples, including those from patients with low sperm counts, the assay was optimised for a lower sperm DNA input and Illumina MiSeq sequencing was used as read-out to facilitate the quantification of all possible nucleotide changes at *FGFR2* c.752–755 (Fig. 1A; see Materials and Methods). This allowed us to estimate the assay background associated with each nucleotide change (Fig. 1A; Supplementary Fig. S2). An additional 26 samples (including three pre-treatment samples) from 16 cancer patients were analysed using this method. Samples were stratified into four groups according to the type of treatment (chemotherapy and/or radiotherapy) and the sterilizing impact of the specific regimens received by patients (Da Cunha *et al.* 1984, Meistrich *et al.* 1990, Meistrich *et al.* 1992, Pryzant *et al.* 1993, Zheng *et al.* 2000, Dubey *et al.* 2000, May *et al*. 2000, Meistrich, 2013). The time to recovery of spermatogenesis for patients treated with alkylating agents was used as an estimate of the gonadotoxic impact of the treatments (Table I, Supplementary Tables SI and SII, Supplementary Fig. S1).

**Figure 2:**
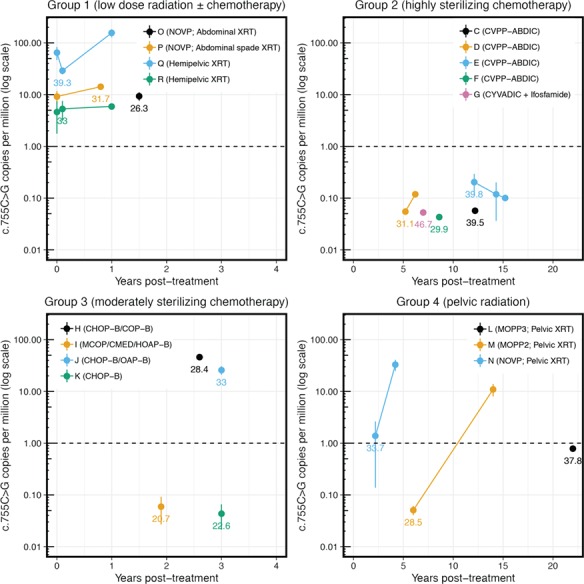
***FGFR2* c.755C > G mutation levels relative to time post-treatment.** Patients have been stratified by treatment regimens (see Table I); Points represent mean measurements; vertical lines represent standard error of the mean (where applicable). For each group, different samples from a single individual have been assigned a colour (key on graph) and are connected by matching coloured lines. Ages at first timepoint post-treatment are plotted adjacent to the data point. Note that the scale of the *x*-axis differs across the groups. The dashed line (1 cpm) represents the estimated background of the assay (Goriely *et al.* 2003). XRT = Radiotherapy.

Group 1 comprises four patients (O-R) who underwent radiation treatment (low scattered gonadal doses of 0.35–0.65 Gy) either as monotherapy for testicular cancer, or in combination with non-alkylating chemotherapy (NOVP) for Hodgkin’s lymphoma (Table I, Fig. 2). Pre-treatment samples were available for three patients (P, Q, R) and these showed c.755C > G mutation levels of 9, 65 and 5 cpm, respectively, consistent with levels measured in untreated individuals of the same age group (30–40 years). While patients P and R showed no notable changes in mutation level 1 year post-treatment, patient Q’s levels increased approximately two-fold over the same period. For Patient O only one post-treatment sample was available at 1.5 years post-treatment and it was in the normal range for his age (9 cpm).

Most of the patients who underwent treatment with highly-sterilizing regimens of alkylating agents (Group 2: patients C-G) were azoospermic or severely oligospermic for ~5 years, so semen samples were only available for analysis 5 to 15 years post-treatment (Supplementary Figs. S1 and S3, Supplementary Table SII). In contrast to those receiving less sterilizing therapy, all Group 2 patients had low background levels of c.755C > G mutations after treatment (Fig. 2), despite being at an age when c.755C > G mutations are detected in most untreated men (Fig. 1B, left panel).

Group 3 consists of four patients (H-K) treated for non-Hodgkin’s lymphoma with CHOP or a similar regimen including moderately-sterilizing doses of cyclophosphamide. Consistent with the milder gonadotoxic impact of their treatment, patients in this group recovered to normospermic levels <3 years after end of treatment (Pryzant *et al.* 1993). Only one sample per patient (obtained between 2 and 3 years after therapy) and no pre-treatment samples were available for analysis in this group (Supplementary Figs. S1 and S3, Supplementary Tables SII, SIV). While two of the individuals (H & J) had c.755C > G levels similar to controls, the other two younger patients (I & K) exhibited background mutation levels (Fig. 2).

Lastly, we analysed Group 4, consisting of three patients (L-N) treated with highly-sterilizing gonadal doses (1.9–4.2 Gy) of pelvic radiotherapy and either moderate doses of alkylating (MOPP) or non-alkylating (NOVP) agents (Supplementary Table SI). At the first time point post-treatment, all three had background levels of c.755C > G mutations (Fig. 2). Patient N (NOVP, 1.9 Gy) showed low mutation levels at 2.2 years post-treatment, but the mutation prevalence increased to 33 cpm 2 years later. Patient M (two courses of MOPP, 3.1 Gy), exhibited background levels of c.755C > G mutations 6 years post-therapy, but 8 years later (14 years post-treatment) his sperm mutation levels had increased to 11 cpm. Finally, for patient L (three courses of MOPP, 4.1 Gy) the single sample available still showed background levels (0.8 cpm) 22 years post-treatment, possibly as a result of his higher treatment doses (Table I and Supplementary Table SI).

**Figure 3:**
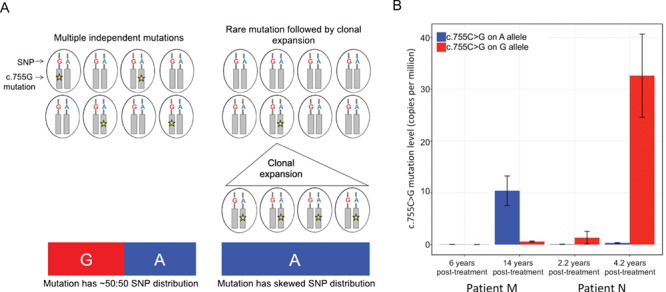
**Phasing of *FGFR2* c.755C > G mutations in heterozygous samples in respect to the rs2071616G > A single nucleotide polymorphism.** (**A**) Schematic explaining that in heterozygous individuals, mutations can arise randomly on either the A or G allele of the *FGFR2* single nucleotide polymorphism (SNP) rs2071616. If mutation levels are high due to multiple independent mutations occurring randomly on either allele, the phasing of the SNP in the c.755C > G mutant cells is likely to be ~50:50. If the mutation levels are high due to clonal expansion of a single mutant cell, the mutation phase should be restricted to a single allele of the SNP. (**B**) Mutation levels in heterozygous samples M and N increase over time and are predominantly phased to a single allele.

Since patients M and N are heterozygous for a naturally occurring SNP (rs2071616G > A) located 118 bp upstream of c.755 (Fig. 1A), we were able to assign the c.755C > G mutation counts to one or the other *FGFR2* allele and, in doing so, determine the likely contribution of independent mutational events to the overall c.755C > G mutational load in these patients (Fig. 3A). When the c.755C > G mutation levels increased to a detectable range above background, we observed that for patient M, 94.3 ± 1.6% were phased to the rs2071616-A allele. Similarly, for patient N (sample N2 taken 4.2 years post-treatment), 99.0 ± 0.3% of the 755C > G mutations were phased with the rs2071616-G allele (Fig. 3B). These results indicate that the sperm carrying the c.755C > G mutations post-treatment in these two patients are derived from a single (or very few) mutant clone(s) of SSCs.

The levels of the c.755C > T mutation (associated with mild Crouzon syndrome) in patient sperm samples were generally below background levels of the assay for this transition (~10 cpm) (Supplementary Figs. S2 and S4). Detectable levels of c.755C > T were only observed in patients’ Q and R pre-treatment samples with similar levels post-treatment, and in the single post-treatment sample of patient J. Of note patient Q, who had three samples with detectable c.755C > T mutations, also had the highest level of c.755C > G mutations. Substitutions not associated with selection (c.755C > A and all mutations at c.752–754) were present at or below background levels of the assay (Supplementary Fig. S2).

## Discussion

Following cancer therapy, recovery of spermatogenesis is believed to occur through expansion and repopulation of the niche by SSCs that have survived treatment (Kanatsu-Shinohara *et al*. 2003, Meistrich 2013). Therefore, the genetic integrity of the surviving cells is of key importance. In this study we assessed spontaneous mutation levels at a known disease-associated genomic location in the sperm of cancer survivors, using samples collected before and up to 22 years after treatment. In the normal context, these selfish mutations confer a selective advantage to SSCs, which leads to clonal expansion of mutant cells and results in an age-related increase in mutation levels in sperm (Fig. 1B). In patients with mildly-sterilizing treatments (Group 1), whose sperm count recovered <1.1 years post-treatment, Apert syndrome mutation (*FGFR2* c.755C > G) levels were in the normal range when compared to age-matched controls and did not change markedly between pre- and post-treatment measurements. As treatments with low gonadotoxic impact are unlikely to have eradicated the majority of SSCs, our data suggest that *FGFR2* Apert-mutant SSCs also survived the treatment, as the relative proportion of sperm derived from mutant clones remained unchanged after recovery from treatment. Two of the four patients treated with moderately-sterilizing therapy (Group 3), whose sperm count recovered within 3 years, exhibited normal age-appropriate mutation levels, but two (I & K) had background levels of mutations. However, as the latter patients were young (treated before the age of 20 years and assessed at 20.7 and 22.6 years), it is plausible that these individuals may have initially harboured few (or no) pre-existing *FGFR2*-mutant SSCs.

Patients with highly-sterilizing treatments (recovery >5 years post-treatment; two patients analysed by pyrosequencing, Groups 2 and 4) had Apert mutation levels at or below the background (~1 cpm) following initial recovery of the sperm count. This was unexpected given their age (Figs 1B and 2). Moreover, in the two patients with pre-treatment samples (A & B), we could demonstrate that mutation levels at this timepoint were in the normal range for their age and had decreased post-treatment (Fig. 1B, left panel). Paradoxically, our data indicate that highly-sterilizing treatments appear to lower the risk of ‘selfish’ genetic disease in the progeny of these men. We propose that this could be explained by the low abundance of selfish Apert syndrome mutations in untreated men, which are only present in a minority of SSCs (~10^−4^ to 10^−6^). Hence, upon highly-sterilizing treatments where the vast majority of SSCs are lost, the likelihood that such mutant SSCs survive would be low. However, given that *FGFR2* c.755C > G encodes a gain-of-function mutation that is oncogenic in other cellular contexts (Dutt *et al.* 2008) and confers a proliferative/growth advantage to mutant SSCs (Goriely *et al.* 2003, Goriely and Wilkie 2012, Martin *et al.* 2014), we speculate that the SSCs carrying such selfish mutations may be preferentially eradicated by strong chemotherapy treatments. Although a few samples had detectable c.755C > T mutations (encoding the milder gain-of-function p.S252L) above background level, all other substitutions were below the background levels of the assay (Supplementary Fig. S2).

In three patients (B, M, N), we observed that selfish clones likely emerged after initially detecting background levels of mutations. In patients M and N (Group 3), the c.755C > G mutation levels markedly increased (>20-fold) within 8 and 2 years, respectively. This increase is much more rapid than the apparent spontaneous increase in c.755C > G levels observed in untreated individuals, which across a population of adult males is estimated to be 10–100-fold over 40 years (Goriely *et al.* 2003). In these two patients, the increase in mutation levels (>20-fold) vastly exceeds the relative increase in sperm count (1.5–3.5-fold) (Table I; Supplementary Fig. S1 and Supplementary Table SII) suggesting a bias in repopulation of the niche by *FGFR2*-mutant stem cells (either by outcompeting wild-type stem cells via preferential growth/survival, or through neutral drift). Consistent with this interpretation, the strong bias of the c.755C > G mutations to a single *FGFR2* allele (based on the phasing of the mutation counts in respect to a nearby heterozygous SNP) suggests that the emerging clones had a single, or very few, mutational origin(s) (Fig. 3). For patient B, spermatogenesis had recovered (110 million/ml) when measured 8.5 years post-treatment at which time point mutations were undetectable: however, 4 years later when sperm count increased to 380 million/ml, a moderate increase in levels of the milder c.755C > T mutation was detected.

Although data from animal models demonstrate an increase of birth defects and mutations in the offspring of males previously exposed to chemotherapy and radiotherapy (Witt and Bishop 1996), our direct assessment of spontaneous mutations at the *FGFR2* locus complement the published human epidemiological and trio WGS data, which so far found no evidence for a substantial long-term mutagenic effect in human sperm. This discrepancy may be explained by differences in SSC dynamics between rodents and humans (Dym *et al.* 2009), differences in treatment regimens, timing post-exposure, or because the rodent assays are more sensitive due to homogeneous genetic backgrounds. Although mutations at *FGFR2* c.755 are a representative model for selfish mutations in general, further studies will be required to confirm the findings in other genes such as *FGFR3* (associated with achondroplasia and thanatophoric dysplasia) and *HRAS* (associated with Costello syndrome).

In conclusion, using an ultra-sensitive assay, we show that gonadotoxic therapy does not lead to an overall increase in the levels of neutral or selfish *FGFR2* mutations in human sperm. Although for a few individuals a notable increase was observed over time, levels were in the same range as age-matched untreated men. Most surprisingly, highly-sterilizing treatments paradoxically appear to reduce the mutational burden associated with age-related clonal selfish expansion. Our data on selfish *FGFR2* mutations complement the body of evidence from epidemiological studies and experimental data in humans suggesting that male germline stem cells are resilient to the accumulation of spontaneous mutations. While this work was performed on samples from only 18 patients who were exposed to different treatment regimens, we hope that our results will encourage others with larger collections of sperm samples from treated patients to replicate and broaden our study. Such data are valuable as they provide physicians and health-care professionals with experimental-based evidence to support counselling of male cancer patients contemplating their reproductive options several years after treatment.

## Supplementary Material

Supp_S1_dez090

Supp_S2_dez090

Supp_S3_dez090

supplementary_data_figure_s4_dez090

Supp_Table1_dez090

Supp_Table2_dez090

Supp_Table3_dez090

Supp_Table4_dez090
