# Supplementary material for: The impact of chemo- and radiotherapy treatments on selfish de novo FGFR2 mutations in sperm of cancer survivors
Source: Hum Reprod. 2019 Jul 26;34(8):1404–15. doi: 10.1093/humrep/dez090 (PMC6688873; doi:10.1093/humrep/dez090)
Supplement: Supp_S1_dez090 [file supp_s1_dez090.pdf]

Human Reproduction, pp. 1–1, 2018
doi:10.1093/humrep/dez090

human reproduction

SUPPLEMENTARY DATA

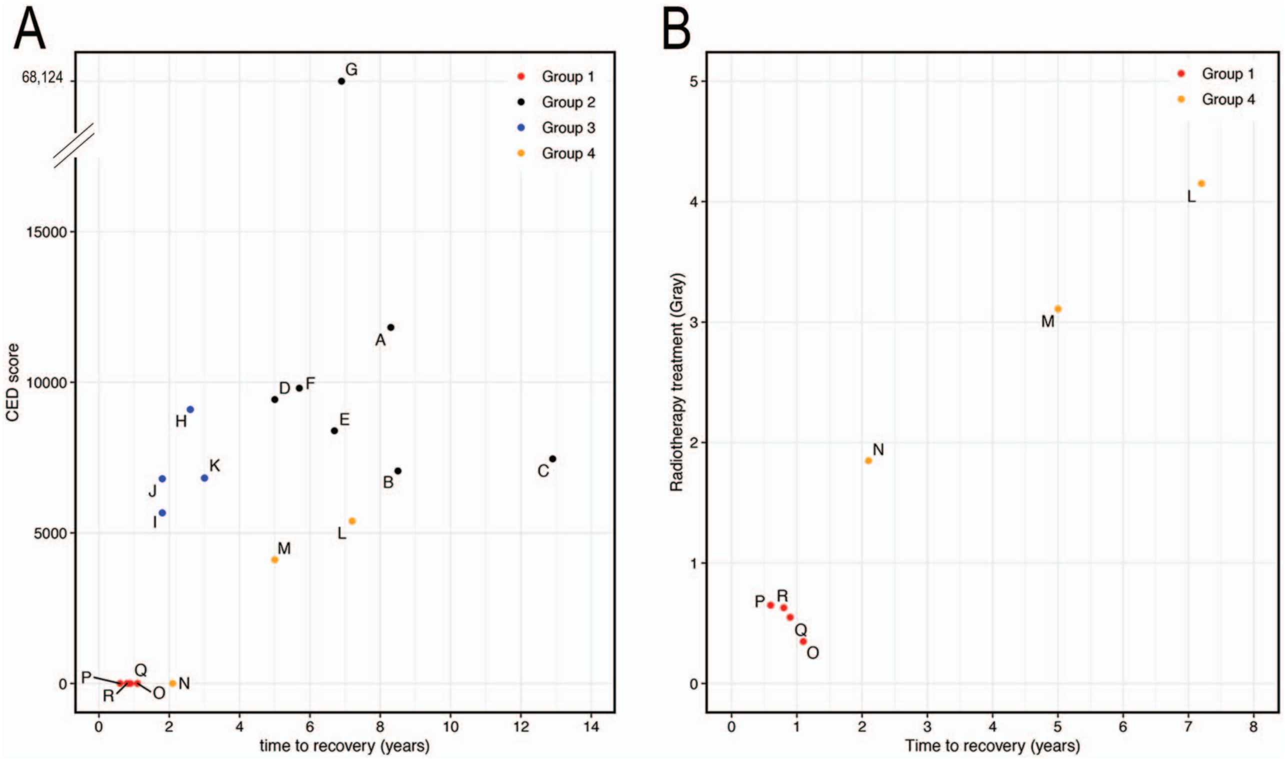

Supplementary Figure S1 Time to spermatogenesis recovery in each of the treatment groups. (A) Time to recovery (years) is plotted against the cyclophosphamide equivalent dose (CED) score (mg/m2). While patients in Groups 2 and 3 were treated with alkylating agents and have overlapping CED scores, they can be further stratified according to their recovery times (>5 years for patients in Group 2 and < 3 years for patients in Group 3). **(B)** For patients treated with radiotherapy (Groups 1 and 4), time to recovery (years) is plotted against the cumulative radiation dose (Gray). Patients in Group 1 who received doses <1 Gy, recovered faster (<2 years) than patients in Group 4 who were exposed to higher doses (>1.8 Gy).
