# Supplementary material for: The impact of chemo- and radiotherapy treatments on selfish de novo FGFR2 mutations in sperm of cancer survivors
Source: Hum Reprod. 2019 Jul 26;34(8):1404–15. doi: 10.1093/humrep/dez090 (PMC6688873; doi:10.1093/humrep/dez090)
Supplement: Supp_S2_dez090 [file supp_s2_dez090.pdf]

Human Reproduction, pp. 1–1, 2018
doi:10.1093/humrep/dez090

human reproduction

SUPPLEMENTARY DATA

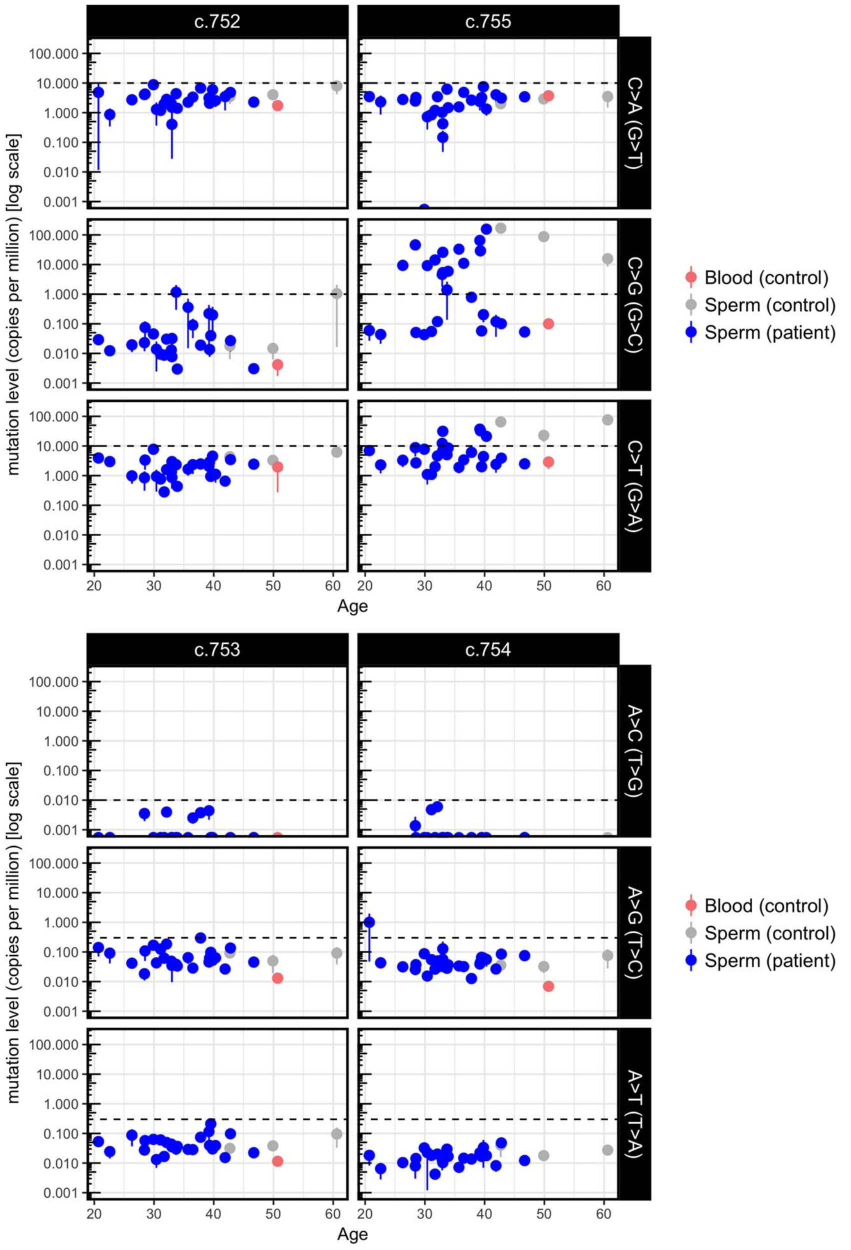

Supplementary Figure S2 **Mutation levels for all possible substitutions at *FGFR2* c.752-c.755.** **(A)** The sequence contexts of *fibroblast growth factor receptor 2* (*FGFR2*) c.752 and c.755 are identical, facilitating comparison of mutation levels at non-selected (c.752, c.755C > A) and selected (c.755C > G, c.775C > T) substitutions. Background thresholds (dashed line) are based on levels at non-selected sites. **(B)** The sequence contexts of c.753 and c.754 are identical, thus demonstrating similar background levels (dashed lines).
