# Supplementary material for: The impact of chemo- and radiotherapy treatments on selfish de novo FGFR2 mutations in sperm of cancer survivors
Source: Hum Reprod. 2019 Jul 26;34(8):1404–15. doi: 10.1093/humrep/dez090 (PMC6688873; doi:10.1093/humrep/dez090)
Supplement: Supp_S3_dez090 [file supp_s3_dez090.pdf]

Human Reproduction, pp. 1–1, 2018
doi:10.1093/humrep/dez090

human reproduction

SUPPLEMENTARY DATA

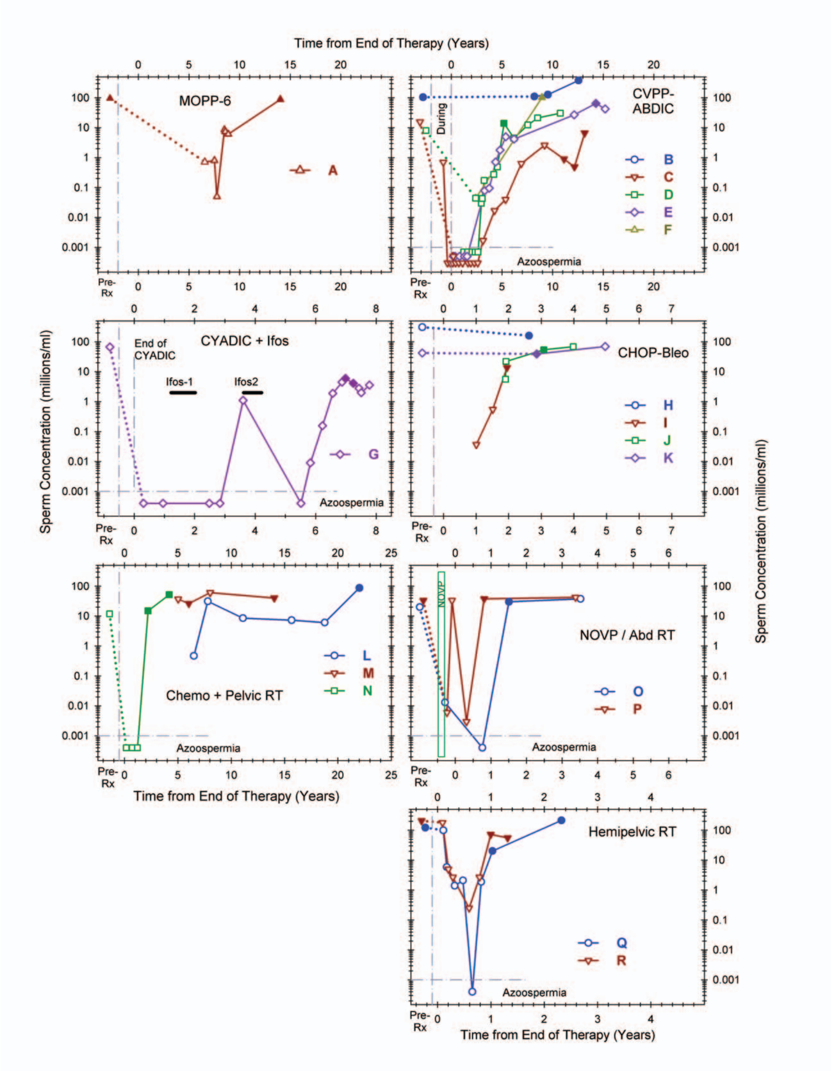

Supplementary Figure S3 **Sperm counts of patients analysed for *FGFR2* mutations**. Open symbols indicate samples that were counted but not analysed for mutations; filled symbol indicate samples that were analysed for mutations. Patient G had long periods of time of remission after CYADIC (cyclophosphamide, doxorubicin, dacarbazine) and after the first ifosfamide (IFOS) treatment but subsequently had recurrences. The end of the second ifosfamide treatment is considered as the end of therapy. Patients O and P had sperm counts and the end or after NOVP (Novantrone, Oncovin, Velban, prednisone) chemotherapy. They subsequently received abdominal radiotherapy, the final treatment of which is considered as the end of therapy. Different scales along the *x*-axis are used for different panels.
