# Supplementary material for: The impact of chemo- and radiotherapy treatments on selfish de novo FGFR2 mutations in sperm of cancer survivors
Source: Hum Reprod. 2019 Jul 26;34(8):1404–15. doi: 10.1093/humrep/dez090 (PMC6688873; doi:10.1093/humrep/dez090)
Supplement: supplementary_data_figure_s4_dez090 [file supplementary_data_figure_s4_dez090.pdf]

Human Reproduction, pp. 1–1, 2018
doi:10.1093/humrep/dez090

human reproduction

SUPPLEMENTARY DATA

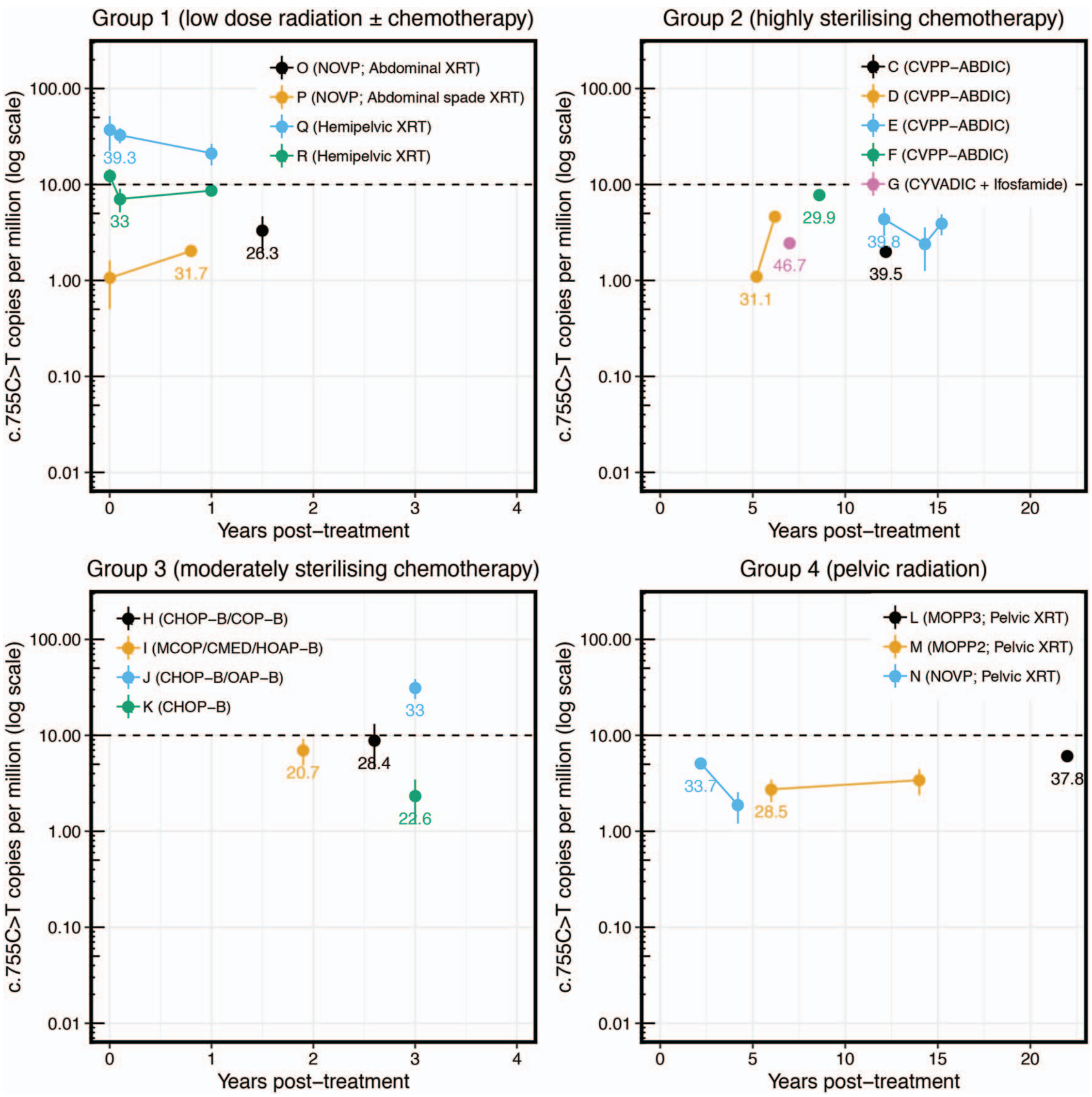

Supplementary Figure S4 ***FGFR2*** **c.755C > T mutation levels relative to time post-treatment**. Patients are grouped by treatment categories (see Table I); Points represent mean measurements; vertical lines represent standard error of the mean (where applicable). For each group, different samples from a single individual have been assigned a colour (key on graph) and are connected by matching coloured lines. Ages at first timepoint post-treatment are plotted adjacent to the data point. Note that the scale of the *x*-axis differs across the groups. The dashed line (10 cpm) represents the estimated background of the assay (Goriely *et al.* 2003). XRT = Radiotherapy.
