# Supplementary material for: The impact of chemo- and radiotherapy treatments on selfish de novo FGFR2 mutations in sperm of cancer survivors
Source: Hum Reprod. 2019 Jul 26;34(8):1404–15. doi: 10.1093/humrep/dez090 (PMC6688873; doi:10.1093/humrep/dez090)
Supplement: Supp_Table1_dez090 [file supp_table1_dez090.pdf]

Supplementary Table SI Detailed patient treatment regimens.

Group ID	Patient	Diagnosis	Chemotherapy/ [Radiation field]	Cycles chemo	Included in publications#	HN2 (mg/m2)	PCZ (mg/m2)	CP (mg/m2)	CCNU (mg/m2)	IFOS (g/m2)	CED¶ (mg/m2)	DOX (mg/m2)	Gonadal Radiation (Gy)‡	Time to recovery
2	A	Hodgkin's IIIA	MOPP6	6	Brandriff, 1994<br>Zheng, 2000	72	5400				11 828		0	~8.3
2	B	Hodgkin's—IIB	CVPP-ABDIC	3 + 3	Zheng, 2000		2822	2806	115		7065	150	<0.05	<8.5
2	C	Hodgkin's—IVB	CVPP-ABDIC	3 + 5	Meistrich, 2013		3000	2689	138		7467	208	<0.05	~12.9
2	D	Hodgkin's—IVB	CVPP-ABDIC	4 + 4	Meistrich, 2013		3092	3827	185		9437	189	<0.05	~5.0
2	E	Hodgkin's—CSIA, relapse	CVPP-ABDIC	4 + 4	Meistrich, 2013		3189	3309	147		8398	189	<0.05	~6.7
2	F	Hodgkin's—IVB	CVPP-ABDIC	4 + 4	(Meistrich, 2013)		4019	3711	166		9809	223	0	5.6 (2.7–8.7)
2	G	Synovial cell sarcoma	CYADIC+Ifosfamide	11 + 19	Meistrich, 1992			8100		246	68 124	928	<0.05	6.9
3	H	NHL—DLCL IEA	CHOP-B/COP-B	9 + 3	Pryzant, 1993			9100			9100	460	<0.05	<2.6
3	I	NHL—Burkitts	MCOP/CMED/ HOAP-B	5 + 2 + 4	Pryzant, 1993			5674			5674	139	0	~1.8
3	J	NHL—Follicular mixed cell	CHOP-B/OAP-B	9 + 9	Pryzant, 1993			6800			6800	450	0	~1.8
3	K	NHL—LCL	CHOP-B	12	(Pryzant, 1993)			6829			6829	304	<0.05	<3
4	L	Hodgkin's—IIIA	MOPP3/[Pelvic]	3	DaCunha, 1984	36	2100				5400		4.15(calc)	7.2
4	M	Hodgkin's—IIA	MOPP2/[Pelvic]	2	Brandriff, 1994	24	2000				4114		3.11(calc)	<5
4	N	Hodgkin's—IIA	NOVP/[Pelvic]	3	Dubey, 2000						0		1.85(calc)	2.1
1	O	Hodgkin's—IIA	NOVP/[Abdominal]	3	Dubey, 2000						0		0.35(TLD)	~1.1
1	P	Hodgkin's—IIIA	NOVP/[Abdominal spade]	3	Dubey, 2000						0		0.65(other pts)	~0.6
1	Q	Seminoma—I	[Hemipelvic]		(May, 2000)						0		0.55(TLD)	0.9
1	R	Seminoma—I	[Hemipelvic]		(May, 2000)						0		0.63(TLD)	0.8

Drugs and abbreviations: NHL: Non-Hodgkin's lymphoma, HN2, nitrogen mustard; PCZ, procarbazine; CP, cyclophosphamide; CCNU, chloroethyl-cyclohexyl-nitrosourea (lomustine); IFOS, ifosfamide; CED (Cyclophosphamide Equivalent Dose); DOX (Doxorubicin/Adriamycin). Regimens: MOPP (HN2, Oncovin, PCZ, prednisone); CVPP-ABDIC (CP, vinblastine, PCZ, prednisone, DOX, bleomycin, dacarbazine, CCNU); CYADIC (CP, DOX, dacarbazine); CHOP-B (CP, DOX, Oncovin, prednisone, bleomycin); MCOP (Mitoxantrone [Novantrone], CP, Oncovin, prednisolone); CMED (CP, methotrexate, etoposide, dexamethasone); HOAP-B (DOX, Oncovin, Cytarabine, prednisone, bleomycin); NOVP (Mitoxantrone/Novantrone, Oncovin, Velban, prednisone).

#Patients whose sperm count or genetic analysis of sperm has been included in previous publications are listed. Those listed in parentheses were not included in the publication, but received similar treatments.

¶ Cyclophosphamide equivalent dose calculated according to Green et al. (2014)

‡Determinations of radiation dose: calc, Calculated scattered gonadal radiation from actual fields and doses for this patient; TLD, measured with thermoluminescent dosimeter placed on scrotum; other pts: based on TLD measurements of patients treated with similar radiation fields.

*See Supplementary Table SII for further details
