# Supplementary material for: The impact of chemo- and radiotherapy treatments on selfish de novo FGFR2 mutations in sperm of cancer survivors
Source: Hum Reprod. 2019 Jul 26;34(8):1404–15. doi: 10.1093/humrep/dez090 (PMC6688873; doi:10.1093/humrep/dez090)
Supplement: Supp_Table2_dez090 [file supp_table2_dez090.pdf]

Human Reproduction, pp. 1–4, 2018
doi:10.1093/humrep/dez090

human reproduction

SUPPLEMENTARY DATA

Supplementary Table SII Detailed sperm counts and volumes of each collected sample for the 18 patients analysed in this study.

Patient ID	Diagnosis	Chemotherapy/[Radiation field] (See also Table S1	Sample ID	Stage of treatment	Age (years)	Time post treatment (years)	Million sperm per ml	Volume of ejaculate (ml)
A	Hodgkin's IIIA	MOPP6	A0	Pre	20.7	Pre	100	2.2
A	Hodgkin's IIIA	MOPP6		Pre	20.7	Pre	168	2.0
A	Hodgkin's IIIA	MOPP6		Pre	20.7	Pre	100	1.2
A	Hodgkin's IIIA	MOPP6		Pre	20.7	Pre	50	1.8
A	Hodgkin's IIIA	MOPP6		Post	27.7	6.6	0.7	3.8
A	Hodgkin's IIIA	MOPP6		Post	28.6	7.5	0.8	4.2
A	Hodgkin's IIIA	MOPP6		Post	28.9	7.8	0.1	ND
A	Hodgkin's IIIA	MOPP6		Post	29.6	8.5	8.6	1.8
A	Hodgkin's IIIA	MOPP6		Post	29.7	8.6	7.3	3.5
A	Hodgkin's IIIA	MOPP6		Post	30.0	8.9	6.1	ND
A	Hodgkin's IIIA	MOPP6	A1	Post	35.2	14.0	87	4.3
B	Hodgkin's—IIB	CVPP-ABDIC	B0	Pre	18.8	Pre	168	1.0
B	Hodgkin's—IIB	CVPP-ABDIC		Pre	18.8	Pre	180	1.5
B	Hodgkin's—IIB	CVPP-ABDIC		Pre	18.8	Pre	37	1.5
B	Hodgkin's—IIB	CVPP-ABDIC	B1	Post	27.8	8.5	110	2.8
B	Hodgkin's—IIB	CVPP-ABDIC	B2	Post	28.8	9.5	127	1.9
B	Hodgkin's—IIB	CVPP-ABDIC	B3	Post	31.8	12.6	380	1.3
C	Hodgkin's—IVB	CVPP-ABDIC		During	26.6	−0.8	0.7	2.0
C	Hodgkin's—IVB	CVPP-ABDIC		During	27.0	−0.4	0	2.3
C	Hodgkin's—IVB	CVPP-ABDIC		During	27.3	−0.1	0	1.6
C	Hodgkin's—IVB	CVPP-ABDIC		Post	27.5	0.1	0	1.4
C	Hodgkin's—IVB	CVPP-ABDIC		Post	27.8	0.4	0	2.1
C	Hodgkin's—IVB	CVPP-ABDIC		Post	28.1	0.7	0	2.1
C	Hodgkin's—IVB	CVPP-ABDIC		Post	28.5	1.1	0	2.8
C	Hodgkin's—IVB	CVPP-ABDIC		Post	29.0	1.6	0	4.0
C	Hodgkin's—IVB	CVPP-ABDIC		Post	29.3	1.9	0	1.8
C	Hodgkin's—IVB	CVPP-ABDIC		Post	29.6	2.3	0	2.2
C	Hodgkin's—IVB	CVPP-ABDIC		Post	30.0	2.6	0	3.2
C	Hodgkin's—IVB	CVPP-ABDIC		Post	30.5	3.1	0.002	3.0
C	Hodgkin's—IVB	CVPP-ABDIC		Post	31.6	4.3	0.017	2.0
C	Hodgkin's—IVB	CVPP-ABDIC		Post	32.7	5.4	0.04	3.5
C	Hodgkin's—IVB	CVPP-ABDIC		Post	34.3	6.9	0.6	1.8
C	Hodgkin's—IVB	CVPP-ABDIC		Post	36.6	9.2	2.6	2.1
C	Hodgkin's—IVB	CVPP-ABDIC	C1	Post	38.5	11.1	0.9	1.6
C	Hodgkin's—IVB	CVPP-ABDIC	C2	Post	39.5	12.2	0.5	5.0
C	Hodgkin's—IVB	CVPP-ABDIC	C3	Post	40.5	13.2	6.7	2.1
D	Hodgkin's—IVB	CVPP-ABDIC		Post	27.2	1.2	0	0.5
D	Hodgkin's—IVB	CVPP-ABDIC		Post	27.6	1.6	0	3.6

Supplementary Table SII *Continued*

Patient ID	Diagnosis	Chemotherapy/[Radiation field] (See also Table S1	Sample ID	Stage of treatment	Age (years)	Time post treatment (years)	Million sperm per ml	Volume of ejaculate (ml)
D	Hodgkin's—IVB	CVPP-ABDIC		Post	28.0	2.1	0	0.5
D	Hodgkin's—IVB	CVPP-ABDIC		Post	28.3	2.4	0	3.6
D	Hodgkin's—IVB	CVPP-ABDIC		Post	28.6	2.6	0	1.5
D	Hodgkin's—IVB	CVPP-ABDIC		Post	28.9	3.0	0.03	1.8
D	Hodgkin's—IVB	CVPP-ABDIC		Post	29.0	3.1	0.04	2.8
D	Hodgkin's—IVB	CVPP-ABDIC		Post	29.2	3.2	0.2	2.9
D	Hodgkin's—IVB	CVPP-ABDIC		Post	30.1	4.2	0.3	1.6
D	Hodgkin's—IVB	CVPP-ABDIC		Post	30.5	4.5	0.5	3.1
D	Hodgkin's—IVB	CVPP-ABDIC	D1	Post	31.1	5.2	14	2.6
D	Hodgkin's—IVB	CVPP-ABDIC	D2	Post	32.1	6.2	4.5	3.2
D	Hodgkin's—IVB	CVPP-ABDIC	D3	Post	33.5	7.6	12.3	2.2
D	Hodgkin's—IVB	CVPP-ABDIC	D4	Post	34.5	8.5	21	2.4
D	Hodgkin's—IVB	CVPP-ABDIC	D5	Post	36.7	10.8	31	2.1
E	Hodgkin's—CSIA, relapse	CVPP-ABDIC		Pre	27.1	Pre	15.5	2.8
E	Hodgkin's—CSIA, relapse	CVPP-ABDIC		Post	27.8	0.2	0	1.0
E	Hodgkin's—CSIA, relapse	CVPP-ABDIC		Post	28.0	0.4	0	2.1
E	Hodgkin's—CSIA, relapse	CVPP-ABDIC		Post	28.4	0.8	0	0.8
E	Hodgkin's—CSIA, relapse	CVPP-ABDIC		Post	28.9	1.3	0	0.9
E	Hodgkin's—CSIA, relapse	CVPP-ABDIC		Post	29.2	1.6	0	1.3
E	Hodgkin's—CSIA, relapse	CVPP-ABDIC		Post	30.9	3.3	0.1	1.0
E	Hodgkin's—CSIA, relapse	CVPP-ABDIC		Post	31.4	3.8	0.1	1.6
E	Hodgkin's—CSIA, relapse	CVPP-ABDIC		Post	32.0	4.4	0.7	1.9
E	Hodgkin's—CSIA, relapse	CVPP-ABDIC		Post	32.4	4.8	1.8	2.1
E	Hodgkin's—CSIA, relapse	CVPP-ABDIC		Post	33.0	5.4	5	1.2
E	Hodgkin's—CSIA, relapse	CVPP-ABDIC		Post	33.8	6.2	4.2	1.1
E	Hodgkin's—CSIA, relapse	CVPP-ABDIC		Post	39.8	12.1	27	2.9
E	Hodgkin's—CSIA, relapse	CVPP-ABDIC	E1	Post	41.9	14.3	65	1.8
E	Hodgkin's—CSIA, relapse	CVPP-ABDIC	E2	Post	42.8	15.2	42	1.5
F	Hodgkin's—IVB	CVPP-ABDIC		Pre	20.7	Pre	8	4.0
F	Hodgkin's—IVB	CVPP-ABDIC		Post	23.9	2.7	0.04	4.5
F	Hodgkin's—IVB	CVPP-ABDIC	F1	Post	29.9	8.6	102	7.5
G	Synovial cell sarcoma	CYADIC+Ifosfamide		Pre	39.4	Pre	67	2.5
G	Synovial cell sarcoma	CYADIC+Ifosfamide		Post	40.0	0.3 *	0	3.5
G	Synovial cell sarcoma	CYADIC+Ifosfamide		Post	40.7	1.0	0	2.4

Supplementary Table SII *Continued*

Patient ID	Diagnosis	Chemotherapy/[Radiation field] (See also Table S1	Sample ID	Stage of treatment	Age (years)	Time post treatment (years)	Million sperm per ml	Volume of ejaculate (ml)
G	Synovial cell sarcoma	CYADIC+Ifosfamide		Post	42.2	2.5	0	0.0
G	Synovial cell sarcoma	CYADIC+Ifosfamide		Post	42.6	2.8	0	1.7
G	Synovial cell sarcoma	CYADIC+Ifosfamide		Post	43.3	3.6	1.1	2.1
G	Synovial cell sarcoma	CYADIC+Ifosfamide		Post	45.3	5.5	0	1.9
G	Synovial cell sarcoma	CYADIC+Ifosfamide		Post	45.6	5.8	0.01	ND
G	Synovial cell sarcoma	CYADIC+Ifosfamide		Post	46.0	6.2	0.16	2.0
G	Synovial cell sarcoma	CYADIC+Ifosfamide		Post	46.3	6.6	1.9	1.6
G	Synovial cell sarcoma	CYADIC+Ifosfamide		Post	46.6	6.9	4.5	2.2
G	Synovial cell sarcoma	CYADIC+Ifosfamide	G1	Post	46.7	7.0	6	2.0
G	Synovial cell sarcoma	CYADIC+Ifosfamide	G2	Post	47.0	7.2	4.1	3.2
G	Synovial cell sarcoma	CYADIC+Ifosfamide		Post	47.2	7.4	2.8	2.0
G	Synovial cell sarcoma	CYADIC+Ifosfamide		Post	47.2	7.5	2.0	3.1
G	Synovial cell sarcoma	CYADIC+Ifosfamide		Post	47.5	7.8	3.6	2.2
H	NHL—DLCL IEA	CHOP-B/COP-B		Pre	24.8	Pre	327	2.0
H	NHL—DLCL IEA	CHOP-B/COP-B		Pre	24.8	Pre	292	2.4
H	NHL—DLCL IEA	CHOP-B/COP-B	H1	Post	28.4	2.6	160	2.2
I	NHL—Burkitts	MCOP/CMED/HOAP-B		Post	19.7	1.0	0.04	0.7
I	NHL—Burkitts	MCOP/CMED/HOAP-B		Post	20.2	1.5	0.6	5.3
I	NHL—Burkitts	MCOP/CMED/HOAP-B	I1	Post	20.7	1.9	13.8	3.4
J	NHL—Follicular mixed cell	CHOP-B/OAP-B		Post	32.0	1.9	5.6	3.1
J	NHL—Follicular mixed cell	CHOP-B/OAP-B		Post	32.0	1.9	22	2.7
J	NHL—Follicular mixed cell	CHOP-B/OAP-B	J1	Post	33.0	3.0	54	2.3
J	NHL—Follicular mixed cell	CHOP-B/OAP-B		Post	34.0	4.0	69	2.9
K	NHL—LCL	CHOP-B		Pre	19.0	Pre	42	5.2
K	NHL—LCL	CHOP-B	K1	Post	22.6	3.0	39	4.5
K	NHL—LCL	CHOP-B		Post	24.6	5.0	70	2.7
L	Hodgkin's—IIIA	MOPP3/[Pelvic]		Post	22.3	6.5	0.5	0.0
L	Hodgkin's—IIIA	MOPP3/[Pelvic]		Post	23.6	7.8	31	1.0
L	Hodgkin's—IIIA	MOPP3/[Pelvic]		Post	26.9	11.1	8.6	2.1
L	Hodgkin's—IIIA	MOPP3/[Pelvic]		Post	31.4	15.7	7.3	1.2
L	Hodgkin's—IIIA	MOPP3/[Pelvic]		Post	34.5	18.8	6.1	ND
L	Hodgkin's—IIIA	MOPP3/[Pelvic]	L1	Post	37.8	22.0	87	0.8
M	Hodgkin's—IIA	MOPP2/[Pelvic]		Post	27.5	5.0	37	3.0
M	Hodgkin's—IIA	MOPP2/[Pelvic]	M1	Post	28.5	6.0	26	3.8
M	Hodgkin's—IIA	MOPP2/[Pelvic]		Post	30.5	8.0	61	3.0
M	Hodgkin's—IIA	MOPP2/[Pelvic]	M2	Post	36.5	14.0	40	2.5
N	Hodgkin's—IIA	NOVP/[Pelvic]		Pre	30.8	Pre	8	4.0
N	Hodgkin's—IIA	NOVP/[Pelvic]		Pre	30.9	Pre	35	4.0
N	Hodgkin's—IIA	NOVP/[Pelvic]		Pre	30.9	Pre	15	4.0
N	Hodgkin's—IIA	NOVP/[Pelvic]		During	31.2	−0.3	11.8	5.5
N	Hodgkin's—IIA	NOVP/[Pelvic]		Post	31.6	0.2	0	5.8
N	Hodgkin's—IIA	NOVP/[Pelvic]		Post	32.2	0.7	0	4.8
N	Hodgkin's—IIA	NOVP/[Pelvic]		Post	32.7	1.2	0	4.3

Supplementary Table SII *Continued*

Patient ID	Diagnosis	Chemotherapy/[Radiation field] (See also Table S1	Sample ID	Stage of treatment	Age (years)	Time post treatment (years)	Million sperm per ml	Volume of ejaculate (ml)
N	Hodgkin's—IIA	NOVP/[Pelvic]	N1	Post	33.7	2.2	15	4.6
N	Hodgkin's—IIA	NOVP/[Pelvic]	N2	Post	35.7	4.2	52	4.7
O	Hodgkin's—IIA	NOVP/[Abdominal]	O1	Pre	24.3	Pre	20	1.5
O	Hodgkin's—IIA	NOVP/[Abdominal]		During	24.5	−0.3	0.013	3.8
O	Hodgkin's—IIA	NOVP/[Abdominal]		Post	25.6	0.8	0	2.7
O	Hodgkin's—IIA	NOVP/[Abdominal]	O2	Post	26.3	1.5	30	4.2
O	Hodgkin's—IIA	NOVP/[Abdominal]	O3	Post	28.3	3.5	38	3.8
P	Hodgkin's—IIIA	NOVP/[Abdominal spade]	P1	Pre	30.4	Pre	46	2.2
P	Hodgkin's—IIIA	NOVP/[Abdominal spade]	P2	Pre	30.4	Pre	19	2.6
P	Hodgkin's—IIIA	NOVP/[Abdominal spade]	P3	Pre	30.4	Pre	11	2.2
P	Hodgkin's—IIIA	NOVP/[Abdominal spade]		During	30.6	−0.2	0.006	3.3
P	Hodgkin's—IIIA	NOVP/[Abdominal spade]		During	30.8	−0.1	33	2.1
P	Hodgkin's—IIIA	NOVP/[Abdominal spade]		Post	31.2	0.3	0.003	1.4
P	Hodgkin's—IIIA	NOVP/[Abdominal spade]	P4	Post	31.7	0.8	38	2.5
P	Hodgkin's—IIIA	NOVP/[Abdominal spade]	P5	Post	34.3	3.4	42	1.8
Q	Seminoma—I	[Hemipelvic]	Q1	Pre	39.2	Pre	123	2.4
Q	Seminoma—I	[Hemipelvic]	Q4	Post	39.3	0.1	99	5.5
Q	Seminoma—I	[Hemipelvic]		Post	39.4	0.2	5.9	5.1
Q	Seminoma—I	[Hemipelvic]		Post	39.5	0.3	1.4	4.1
Q	Seminoma—I	[Hemipelvic]		Post	39.7	0.5	2.1	8.1
Q	Seminoma—I	[Hemipelvic]		Post	39.9	0.7	0	5.1
Q	Seminoma—I	[Hemipelvic]		Post	40.0	0.8	1.9	4.2
Q	Seminoma—I	[Hemipelvic]	Q2	Post	40.3	1.0	20	5.7
Q	Seminoma—I	[Hemipelvic]	Q3	Post	41.5	2.3	213	3.2
R	Seminoma—I	[Hemipelvic]	R1	Pre	32.9	−0.1	209	2.1
R	Seminoma—I	[Hemipelvic]	R4	Post	33.0	0.1	179	2.8
R	Seminoma—I	[Hemipelvic]		Post	33.1	0.2	5.0	1.0
R	Seminoma—I	[Hemipelvic]		Post	33.2	0.3	2.7	1.7
R	Seminoma—I	[Hemipelvic]		Post	33.5	0.6	0.3	0.9
R	Seminoma—I	[Hemipelvic]		Post	33.7	0.8	2.7	1.0
R	Seminoma—I	[Hemipelvic]	R2	Post	33.9	1.0	73	1.2
R	Seminoma—I	[Hemipelvic]	R3	Post	34.2	1.3	56	1.5

ND: Volume not determined.
∗ Time after end of CYADIC treatment. Patient subsequently received IFOS (see Supplementary Figure S2)
