# Supplementary material for: The impact of chemo- and radiotherapy treatments on selfish de novo FGFR2 mutations in sperm of cancer survivors
Source: Hum Reprod. 2019 Jul 26;34(8):1404–15. doi: 10.1093/humrep/dez090 (PMC6688873; doi:10.1093/humrep/dez090)
Supplement: Supp_Table3_dez090 [file supp_table3_dez090.pdf]

Supplementary Table SIII Amplification and sequencing primers.

Process	Details	Modification	Forward primer (5′-3′)	Reverse primer (5′-3′)
Amplification	Initial amplification	-	TTACAAGGGTCGCGCTCCAGCA GTCTCC	GGGGCTGGGCATCACTGTAAACCTTG
Amplification	Nested PCR	Biointylated (5′)	ATTCATGGGGCCACAGTGTTATT TCAAAGG	AACCTTGCAGACAAACTCTACGTCTCC
Amplification	Nested PCR (CS-tagged – tags underlined)	-	<u>TACGGTAGCAGAGACTTGGTCT</u> ATTCATGGGGCCACAGTGTTATT TCAAAGG	<u>ACACTGACGACATGGTTCTACA</u>AACCT TGCAGACAAACTCTACGTCTCC
Amplification	Access Array Illumina barcoding primers targeting CS (Fluidigm)	-	CAAGCAGAAGACGGCATACGAG AT[10 nt index] <u>TACGGTAGCAGAGATTGGTCT</u>	AATGATACGGCGACCACCGAGATCT <u>ACACTGACGACATGGTTCTACA</u>
Pyrosequencing	Pyrosequencing	-	CTCCACCAGAGCGAT	
Illumina sequencing	Read 1	locked nucleic acid (LNA) nucleotides are preceded by a + sign	A + CA + CTG + ACGACATGGTTCTACA	
Illumina sequencing	Read 2	locked nucleic acid (LNA) nucleotides are preceded by a + sign	T + AC + GGT + AGCAGAGACTTGGTCT	
Illumina sequencing	Indexing	locked nucleic acid (LNA) nucleotides are preceded by a + sign	A + GAC + CA + AGTCTCTGCTACCGT	
