# Supplementary material for: The impact of chemo- and radiotherapy treatments on selfish de novo FGFR2 mutations in sperm of cancer survivors
Source: Hum Reprod. 2019 Jul 26;34(8):1404–15. doi: 10.1093/humrep/dez090 (PMC6688873; doi:10.1093/humrep/dez090)
Supplement: Supp_Table4_dez090 [file supp_table4_dez090.pdf]

Supplementary Table SIV Mutations levels for each sample replicates for all possible substitutions at *FGFR2* c.752–755.

Donor	Sample	Replicate number (passed QC)	Sample type	Treatment (see Table SI for details)	Age (years)	Years post treat-ment	Total reads	755C > A cpm	755C > G cpm	755C > T cpm	rs2071616 genotype	755C > A cpm (A allele)	755C > A cpm (G allele)	755C > G cpm (A allele)	755C > G cpm (G allele)	755C > T cpm (A allele)	755 T > G cpm (G allele)	rs2071616 G allele (%)
1086	1086	2	Blood (control)	none	50.73	NA	4787	5.98	0.05	3.98	Homozygous	0.01	5.97	0	0.05	0	3.97	
1086	1086	3	Blood (control)	none	50.73	NA	1737	2.61	0.14	0.46	Homozygous	0	2.61	0.14	0	0.03	0.43	
1086	1086	4	Blood (control)	none	50.73	NA	5785	2.6	0.11	4.35	Homozygous	0	2.6	0.09	0.01	0.01	4.35	
24	277	1	Sperm (control)	none	60.57	NA	9387	5.49	8.51	66.54	Homozygous	0.01	5.48	0.02	8.49	0.08	66.46	
24	277	2	Sperm (control)	none	60.57	NA	9118	1.49	22.91	86.06	Homozygous	0	1.49	0.03	22.88	0.11	85.94	
27	727	1	Sperm (control)	none	42.68	NA	9592	1.4	176.62	64.17	Homozygous	0	1.4	0.19	176.43	0.09	64.07	
27	727	2	Sperm (control)	none	42.68	NA	17 590	2.61	160.98	65.85	Homozygous	0	2.61	0.21	160.77	0.1	65.75	
26	856	1	Sperm (control)	none	49.87	NA	12 538	1.95	102.79	41	Heterozygous	0.06	1.89	81.7	21.09	17.25	23.75	
26	856	2	Sperm (control)	none	49.87	NA	7492	1.8	88.62	12.35	Heterozygous	0.99	0.81	75.81	12.81	3.85	8.5	
26	856	4	Sperm (control)	none	49.87	NA	18 914	4.96	69.96	15.04	Heterozygous	2.02	2.95	57.65	12.31	10.54	4.5	
C	C123	1	Sperm (patient)	cvpp-abdic	39.53	12.16	8182	3.22	0.06	2	Homozygous	0	3.22	0	0.05	0.01	1.99	
D	D1	1	Sperm (patient)	cvpp-abdic	31.14	5.2	4088	0.85	0.05	1.1	Heterozygous	0.84	0.01	0.05	0	0.51	0.59	
D	D2	1	Sperm (patient)	cvpp-abdic	32.12	6.18	9529	3.45	0.12	4.64	Heterozygous	0.56	2.89	0.08	0.04	2	2.64	
E	E0	1	Sperm (patient)	cvpp-abdic	39.80	12.1	5981	7.25	0.38	5.49	Homozygous	6.95	0.3	0.36	0.02	5.16	0.34	
E	E0	2	Sperm (patient)	cvpp-abdic	39.80	12.1	4205	8.5	0.16	1.5	Homozygous	7.98	0.52	0.14	0.02	1.4	0.1	
E	E0	3	Sperm (patient)	cvpp-abdic	39.80	12.1	4250	6.81	0.07	6.1	Homozygous	6.3	0.51	0.05	0.02	5.77	0.33	
E	E1	2	Sperm (patient)	cvpp-abdic	41.88	14.28	3208	0.53	0.28	0.32	Homozygous	0.51	0.02	0.13	0.15	0.24	0.08	
E	E1	3	Sperm (patient)	cvpp-abdic	41.88	14.28	5655	6.96	0.03	2.6	Homozygous	6.76	0.2	0.03	0	2.51	0.09	

Continued

Supplementary Table SIV *Continued*

Donor	Sample	Replicate number (passed QC)	Sample type	Treatment (see Table SI for details)	Age (years)	Years post treat-ment	Total reads	755C > A cpm	755C > G cpm	755C > T cpm	rs2071616 genotype	755C > A cpm (A allele)	755C > A cpm (G allele)	755C > G cpm (A allele)	755C > G cpm (G allele)	755C > T cpm (A allele)	755 T > G cpm (G allele)	rs2071616 G allele (%)
E	E1	4	Sperm (patient)	cvpp-abdic	41.88	14.28	9170	4.63	0.05	4.32	Homozygous	4.26	0.36	0.05	0	3.96	0.35	
E	E2	1	Sperm (patient)	cvpp-abdic	42.80	15.19	8126	3.07	0.11	2.96	Homozygous	2.84	0.22	0.1	0.01	2.73	0.22	
E	E2	2	Sperm (patient)	cvpp-abdic	42.80	15.19	9286	3.14	0.1	4.9	Homozygous	2.86	0.29	0.07	0.03	4.44	0.46	
F	F1	1	Sperm (patient)	cvpp-abdic	29.86	8.63	3672	0	0.04	7.75	Homozygous	0	0	0	0.04	0.01	7.75	
G	G12	1	Sperm (patient)	cyvadic	46.72	6.99	5123	3.43	0.05	2.46	Heterozygous	1.23	2.2	0.01	0.04	1.31	1.15	
H	H1	1	Sperm (patient)	chop	28.41	2.62	7014	4.41	52.37	21.72	Homozygous	0	4.41	0.08	52.29	0.02	21.7	
H	H1	2	Sperm (patient)	chop	28.41	2.62	4123	1.86	31.96	3.09	Homozygous	0	1.86	0.03	31.93	0	3.09	
H	H1	3	Sperm (patient)	chop	28.41	2.62	4406	2.5	47.4	5.81	Homozygous	0.03	2.48	0.02	47.38	0.01	5.8	
H	H1	4	Sperm (patient)	chop	28.41	2.62	6705	1.11	51.93	4.53	Homozygous	0	1.11	0.18	51.75	0.08	4.45	
I	I1	1	Sperm (patient)	chop	20.68	1.95	4998	3.36	0.03	4.83	Homozygous	0.23	3.13	0.01	0.02	0.92	3.91	
I	I1	2	Sperm (patient)	chop	20.68	1.95	9490	3.62	0.09	9.19	Homozygous	1.13	2.48	0.04	0.06	3.39	5.8	
J	J1	1	Sperm (patient)	chop	33.03	2.98	6543	0.06	18.41	19.2	Homozygous	0	0.06	0.02	18.4	0.04	19.17	
J	J1	2	Sperm (patient)	chop	33.03	2.98	7456	0.04	34.75	44.65	Homozygous	0	0.04	0.04	34.7	0.08	44.57	
J	J1	3	Sperm (patient)	chop	33.03	2.98	16000	0.34	24.36	29.96	Homozygous	0	0.34	0.03	24.33	0.04	29.92	
K	K1	1	Sperm (patient)	chop	22.61	2.96	1515	0.02	0	1.24	Homozygous	0.01	0.01	0	0	0.01	1.23	
K	K1	2	Sperm (patient)	chop	22.61	2.96	3164	3.17	0.09	5.6	Homozygous	0	3.17	0.03	0.05	0.02	5.57	
K	K1	3	Sperm (patient)	chop	22.61	2.96	6889	5.98	0.08	2.01	Homozygous	0.01	5.97	0.02	0.05	0	2.01	
K	K1	4	Sperm (patient)	chop	22.61	2.96	4389	0.03	0.01	0.49	Homozygous	0	0.03	0	0.01	0.01	0.48	

Continued

Supplementary Table SIV *Continued*

Donor	Sample	Replicate number (passed QC)	Sample type	Treatment (see Table SI for details)	Age (years)	Years post treat-ment	Total reads	755C > A cpm	755C > G cpm	755C > T cpm	rs2071616 genotype	755C > A cpm (A allele)	755C > A cpm (G allele)	755C > G cpm (A allele)	755C > G cpm (G allele)	755C > T cpm (A allele)	755 T > G cpm (G allele)	rs2071616 G allele (%)
L	L1	2	Sperm (patient)	mopp3, pelvic	37.78	22.02	10 719	2.65	0.79	6.08	Homozygous	0.01	2.64	0.01	0.78	0.01	6.07	
M	M1	1	Sperm (patient)	mopp2-pelvic	28.50	6	4921	5.21	0.06	1.31	Heterozygous	1.29	3.92	0.04	0.02	0.25	1.06	
M	M1	2	Sperm (patient)	mopp2-pelvic	28.50	6	7522	1.45	0.03	3.6	Heterozygous	0.03	1.41	0.02	0.02	2.77	0.82	
M	M1	3	Sperm (patient)	mopp2-pelvic	28.50	6	5509	3.15	0.06	3.3	Heterozygous	1.28	1.87	0.03	0.03	2.25	1.04	
M	M2	1	Sperm (patient)	mopp2-pelvic	36.47	14.03	5110	2.05	16.13	0.46	Heterozygous	1.3	0.75	15.52	0.61	0.23	0.22	
M	M2	2	Sperm (patient)	mopp2-pelvic	36.47	14.03	6883	2.67	15.52	3.99	Heterozygous	1.69	0.97	14.71	0.8	0.4	3.59	
M	M2	3	Sperm (patient)	mopp2-pelvic	36.47	14.03	8788	6.94	3.89	5.17	Heterozygous	3.68	3.27	3.65	0.25	2.61	2.55	
M	M2	4	Sperm (patient)	mopp2-pelvic	36.47	14.03	7541	7.71	8.32	4.03	Heterozygous	3.3	4.41	7.7	0.62	2.48	1.55	
N	N1	1	Sperm (patient)	novp-pelvic	33.68	2.2	4617	6.01	0.05	5.04	Heterozygous	2.93	3.08	0.03	0.03	2.8	2.25	
N	N1	2	Sperm (patient)	novp-pelvic	33.68	2.2	8370	7.04	3.88	5.77	Heterozygous	2.87	4.17	0.11	3.77	3.44	2.33	
N	N1	3	Sperm (patient)	novp-pelvic	33.68	2.2	5163	5.61	0.22	4.47	Heterozygous	1.61	4	0.04	0.19	3.55	0.92	
N	N2	1	Sperm (patient)	novp-pelvic	35.66	4.19	7115	2.19	19.48	0.66	Heterozygous	2.06	0.13	0.18	19.3	0.34	0.31	
N	N2	2	Sperm (patient)	novp-pelvic	35.66	4.19	3584	1.45	19.96	1.02	Heterozygous	0.01	1.43	0.23	19.73	0.78	0.24	
N	N2	3	Sperm (patient)	novp-pelvic	35.66	4.19	5327	1.58	52.51	3.66	Heterozygous	0.86	0.72	0.29	52.22	2.26	1.4	
N	N2	4	Sperm (patient)	novp-pelvic	35.66	4.19	8630	1.04	39.79	2.18	Heterozygous	0.89	0.15	0.53	39.26	1.31	0.87	
O	O2	1	Sperm (patient)	novp-abd	26.32	1.51	9799	2.97	6.26	6.89	Heterozygous	1.66	1.31	5.86	0.39	3.07	3.82	93.6%
O	O2	2	Sperm (patient)	novp-abd	26.32	1.51	12 355	3.71	15.96	0.73	Heterozygous	0.25	3.46	15.16	0.8	0.39	0.35	95.0%
O	O2	3	Sperm (patient)	novp-abd	26.32	1.51	13 126	1.21	8.22	1.49	Heterozygous	0.38	0.83	7.67	0.55	1	0.48	93.3%

Continued

Supplementary Table SIV *Continued*

Donor	Sample	Replicate number (passed QC)	Sample type	Treatment (see Table SI for details)	Age (years)	Years post treat-ment	Total reads	755C > A cpm	755C > G cpm	755C > T cpm	rs2071616 genotype	755C > A cpm (A allele)	755C > A cpm (G allele)	755C > G cpm (A allele)	755C > G cpm (G allele)	755C > T cpm (A allele)	755 T > G cpm (G allele)	rs2071616 G allele (%)
O	O2	4	Sperm (patient)	novp-abd	26.32	1.51	7403	1.22	7.96	6.36	Heterozygous	0.2	1.02	7.26	0.7	1.83	4.53	91.2%
O	O2	5	Sperm (patient)	novp-abd	26.32	1.51	5601	4.83	8.3	1.05	Heterozygous	2.83	2.01	7.87	0.43	0.63	0.43	94.8%
P	P1	1	Sperm (patient)	pre-novp-spade	30.37	0	8516	0.02	12.12	0.53	Homozygous	0	0.02	0.01	12.11	0	0.53	
P	P1	2	Sperm (patient)	pre-novp-spade	30.37	0	4284	1.57	11.27	2.18	Homozygous	0	1.57	0	11.26	0.01	2.17	
P	P1	4	Sperm (patient)	pre-novp-spade	30.37	0	7579	0.58	4.19	0.48	Homozygous	0	0.57	0.01	4.18	0.01	0.47	
P	P4	1	Sperm (patient)	novp-spade	31.70	0.8	14 419	1.19	14.23	2.03	Homozygous	0	1.19	0.04	14.19	0.01	2.02	
Q	Q1	3	Sperm (patient)	pre	39.16	0	8530	0.04	30.02	10.33	Homozygous	0.04	0	29.23	0.79	10.03	0.3	
Q	Q1	4	Sperm (patient)	pre	39.16	0	10 330	3.26	73.39	40.17	Homozygous	3.04	0.22	69.61	3.78	38.19	1.98	
Q	Q1	5	Sperm (patient)	pre	39.16	0	12 393	3.8	91.41	60.89	Homozygous	3.56	0.24	87.25	4.16	57.95	2.93	
Q	Q2	1	Sperm (patient)	hemipelvic	40.25	1.03	15 289	1.41	88.47	14.11	Homozygous	1.37	0.04	85.8	2.68	13.55	0.55	
Q	Q2	2	Sperm (patient)	hemipelvic	40.25	1.03	6532	0.8	166.36	35.99	Homozygous	0.8	0	162.82	3.54	35.03	0.96	
Q	Q2	3	Sperm (patient)	hemipelvic	40.25	1.03	6774	0.32	223.65	12.57	Homozygous	0.31	0.01	218.43	5.21	12.36	0.21	
Q	Q2	4	Sperm (patient)	hemipelvic	40.25	1.03	22 953	2.78	146.16	22.01	Homozygous	2.64	0.14	138.46	7.7	20.75	1.25	
Q	Q4	1	Sperm (patient)	hemipelvic	39.33	0.11	12 770	3.42	33.42	26.86	Homozygous	3.23	0.19	31.48	1.93	25.2	1.66	
Q	Q4	3	Sperm (patient)	hemipelvic	39.33	0.11	14 697	1.7	24.57	38.56	Homozygous	1.58	0.13	22.79	1.78	35.73	2.83	
R	R1	1	Sperm (patient)	pre	32.86	0	7712	0.57	3.96	15.37	Homozygous	0.56	0.01	3.84	0.12	14.91	0.46	
R	R1	2	Sperm (patient)	pre	32.86	0	7842	1.57	9.86	13.23	Homozygous	1.52	0.05	9.57	0.29	12.83	0.4	
R	R1	3	Sperm (patient)	pre	32.86	0	7969	0.94	0.03	8.2	Homozygous	0.92	0.03	0.02	0.01	7.91	0.29	

Continued

Supplementary Table SIV *Continued*

Donor	Sample	Replicate number (passed QC)	Sample type	Treatment (see Table SI for details)	Age (years)	Years post treatment	Total reads	755C > A cpm	755C > G cpm	755C > T cpm	rs2071616 genotype	755C > A cpm (A allele)	755C > A cpm (G allele)	755C > G cpm (A allele)	755C > G cpm (G allele)	755C > T cpm (A allele)	755 T > G cpm (G allele)	rs2071616 G allele (%)
R	R2	1	Sperm (patient)	hemipelvic	33.90	1	7804	1.47	5.92	8.63	Homozygous	1.41	0.06	5.68	0.24	8.31	0.32	
R	R4	1	Sperm (patient)	hemipelvic	33.00	0.09	10 501	0.39	7.56	9.01	Homozygous	0.37	0.02	6.85	0.71	8.19	0.82	
R	R4	2	Sperm (patient)	hemipelvic	33.00	0.09	7342	0.45	3.09	5.13	Homozygous	0.41	0.04	2.83	0.25	4.64	0.48	

Continued
